# Plasma NMDAR autoantibody: a new biomarker for the diagnosis of Hirschsprung disease

**DOI:** 10.3389/fped.2025.1514323

**Published:** 2025-02-21

**Authors:** Yulu Lai, Jieting Lu, Yanqing Liu, Jixiao Zeng, Shenwei Huang, Lin Li, Bingtong Wang, Pengfei Wei, Yu Ouyang, Junjian Lv, Wei Zhong, Chaoting Lan, Huimin Xia, Qiuming He

**Affiliations:** ^1^ The First School of Clinical Medicine, Southern Medical University, Guangzhou, Guangdong, China; ^2^ Guangdong Provincial Key Laboratory of Research in Structural Birth Defect Disease, Department of Pediatric Surgery, Guangzhou Women and Children’s Medical Center, Guangdong Provincial Clinical Research Center for Child Health, Guangzhou Medical University, Guangzhou, Guangdong, China; ^3^ Department of Infection Management, Sanya Central Hospital, Sanya, Hainan, China; ^4^ Guangzhou Women and Children’s Medical Center, Liuzhou Hospital, Liuzhou, Guangxi, China

**Keywords:** biomarker, diagnosis, Hirschsprung disease, NMDAR autoantibody, enteric nervous system

## Abstract

**Introduction:**

Hirschsprung Disease (HSCR) is a common congenital intestinal disease in pediatrics. Early diagnosis and treatment after birth alleviate the occurrence of complications. Consequently, we aim to identifiy a biomarker with ease of use, non-invasiveness, and highly accurate for diagnosis.

**Methods:**

Plasma samples were collected from HSCR group, other intestinal disease controls (DC) and healthy controls (HC)**,** while colon samples were collected from HSCR and DC groups. We conducted human neural autoantibody microarray analyses on plasma. The candidate biomarker was further validated using enzyme-linked immunosorbent assay (ELISA) in colon tissue and plasma. The receiver operating characteristic curve (ROC) was used to assess the diagnostic performance of the plasma biomarker.

**Results:**

Microarray analysis revealed that the level of plasma N-methyl-D-Aspartate receptor (NMDAR) autoantibody in HSCR group was significantly higher than those in the HC group (*p* = 0.008). In plasma analyzed cohort, the level of NMDAR autoantibodies in HSCR group (*n* = 38) were significantly elevated compared to both the HC (*n* = 31, *p* < 0.0001) and the DC (*n* = 20, *p* < 0.0001). We further validated the diagnostic efficacy of plasma NMDAR autoantibody, it demonstrated AUCs of 0.96 and 0.81 for diagnosing HSCR when compared to HC and DC.

**Conclusions:**

Plasma NMDAR autoantibody might be served as an efficient, non-invasive biomarker for diagnosing HSCR.

## Introduction

Hirschsprung Disease (HSCR) is a congenital disorder due to lack of enteric neurons in the distal colon (1–3). HSCR primarily necessitates surgical intervention due to its life-threatening complications and persistently high mortality rates (4–6). HSCR diagnosis and management, particularly the preoperative assessments, continue to be complex and challenging.

At present, HSCR presents with nonspecific clinical manifestations, and intraoperative diagnosis relies heavily on histopathological confirmation of aganglionosis in the distal colon. Common preoperative diagnostic adjuncts include barium enema (BE), anorectal manometry (ARM), and rectal biopsy, all of which are invasive or associated with significant discomfort (7–9). Emerging immunostaining markers, such as acetylcholinesterase (AChE) and calretinin, lack large-scale comparative studies and present challenges in preoperative application (8, 10). Consequently, the investigation of non-invasive, high-precision biomarkers holds significant value for the diagnosis of HSCR.

Recent studies have found that neural autoantibodies cause developmental abnormalities and neuronal damage by binding to neuronal surface antigens (11–13), a mechanism similar to the enteric neuronal developmental disorders in HSCR (14, 15). However, it is unclear whether neural autoantibodies that cause neuronal development and damage are involved in the occurrence and development of HSCR.

Here, we conducted plasma human neural autoantibody microarray analysis in HSCR, and this study aims to further explore biomarkers of diagnostic value.

## Patients and study design

### Study subjects

Our study included three groups: HSCR group, DC group diagnosed with anal atresia or intestinal stenosis, and HC group comprising children undergoing routine physical examinations. The inclusion and exclusion criteria for the three groups are as follows.

Inclusion and Exclusion Criteria for HSCR group:

1. Patients who underwent surgical resection of the affected intestinal tissue with histopathological biopsies showing no aganglionosis (lack of ganglion cells).
2. No other congenital anomalies or syndromes present.
3. Not during an acute enteritis phase.

Inclusion and Exclusion Criteria for Group DC group:

1. Patients clinically diagnosed with congenital anal atresia or intestinal stenosis.
2. After surgical resection of the colonic tissue, histopathological biopsies show normal development of ganglion cells in the colonic tissue.
3. Not during an acute enteritis phase.

Inclusion and Exclusion Criteria for HC group:

1. Children diagnosed as healthy during routine physical examinations.
2. No congenital developmental abnormalities.

In this study, the cohort subjected to Human neural autoantibody microarray analysis of plasma is termed the discovery cohort. For the validation of NMDAR autoantibodies in plasma and colon tissue using ELISA, two additional cohorts were independently recruited at different time periods and are defined as independent cohorts. Recruitment for all three cohorts was strictly in accordance with the aforementioned inclusion criteria, with matching of age and gender among the study subjects across different groups.

Discovery cohort: HSCR (*n* = 5), DC (*n* = 5) and HC (*n* = 5) were tested on the Guangzhou AiGene Biotechnology's human neuroautoantibody microarray platform (Supplementary Table S1).

An independent cohort for ELISA validation of colon tissue: HSCR (*n* = 36), DC (*n* = 11) (Supplementary Table S2).

An additional independent cohort for ELISA validation of plasma: HSCR (*n* = 38), DC (*n* = 20) and HC (*n* = 31) (Supplementary Table S3).

All samples were collected from the Guangzhou Women and Children's Medical Center Clinical Sample Repository.

### Human neural autoantibody microarray

Plasma samples were mixed with human neuroautoantibody microarray that composed of over 100 human neural autoantibody probes, and incubated on a shaker at room temperature for 60 min. After washing, the anti-human IgG and anti-human IgM fluorescent secondary antibodies were diluted 1:1000 and incubated. The microarray was imaged by LuxScan 10K-B scanner and the raw data was read using LuxScan 3.0 software.

### Detection of NMDAR autoantibodies in colon tissue and plasma using ELISA

Colon tissues with and without ganglia from the HSCR group were collected during radical surgery, while those from DC group were harvested from the margins of excision in cases of anal atresia and intestinal stricture. After collection, samples were immediately placed in pre-chilled isotonic saline to prevent tissue degradation and swiftly transferred to the laboratory for further processing. In order to preserve the activity of neural autoantibodies to the greatest extent, the samples were quickly frozen in liquid nitrogen post-collection. Frozen samples were stored in an ultra-low temperature freezer at −80°C.

Blood was collected from HSCR group and the DC group during surgery. Plasma for the HC group was obtained from the residual blood of healthy children after physical examinations. 3 ml of fasting peripheral blood samples from participants were collected using EDTA vacuum tubes and immediately centrifuged at 4°C (1,500 × rpm, 20 min) to separate plasma and blood cells. Following an additional centrifugation at 4°C (3,000 × rpm, 15 min), the plasma samples were promptly frozen and stored at −80°C.

We further used the NMDAR antibody ELISA kit (Shanghai Zhenke Biotechnology Co., Ltd. ZK-4316) to measure NMDAR autoantibody levels in the plasma and colon tissue via ELISA. Each sample underwent three tests, and the average was calculated. The testers were completely unaware of the test results and sample grouping.

### Statistical analysis

For microarray analysis, we used the Robust Linear Model method for normalization, followed by cluster analysis after M-statistic screening. The data obtained from ELISA testing were compared between groups using unpaired *t*-tests. Linear correlation analysis was used to evaluate the correlation between NMDAR autoantibody expression level in colon tissue with and without ganglionic segments in HSCR. We assessed the diagnostic efficacy of NMDAR autoantibody using the area under the Receiver Operating Characteristic curve (AUC). The statistical software used was R software (version 4.2.3) and GraphPad Prism 9.5 (GraphPad Software Inc., CA, USA). All *p*-values were two-sided, with *p* < 0.05 deemed to have statistical significance.

### Ethics committee approval

This project was approved by the review committee of Institution Guangzhou Women and Children's Medical Center (No. 2016042036), and the use of clinical data and samples has been consented to in writing by the parents or legal guardians of the participants.

## Results

### Cluster analysis of human neural autoantibody microarray

**Figure 1:**
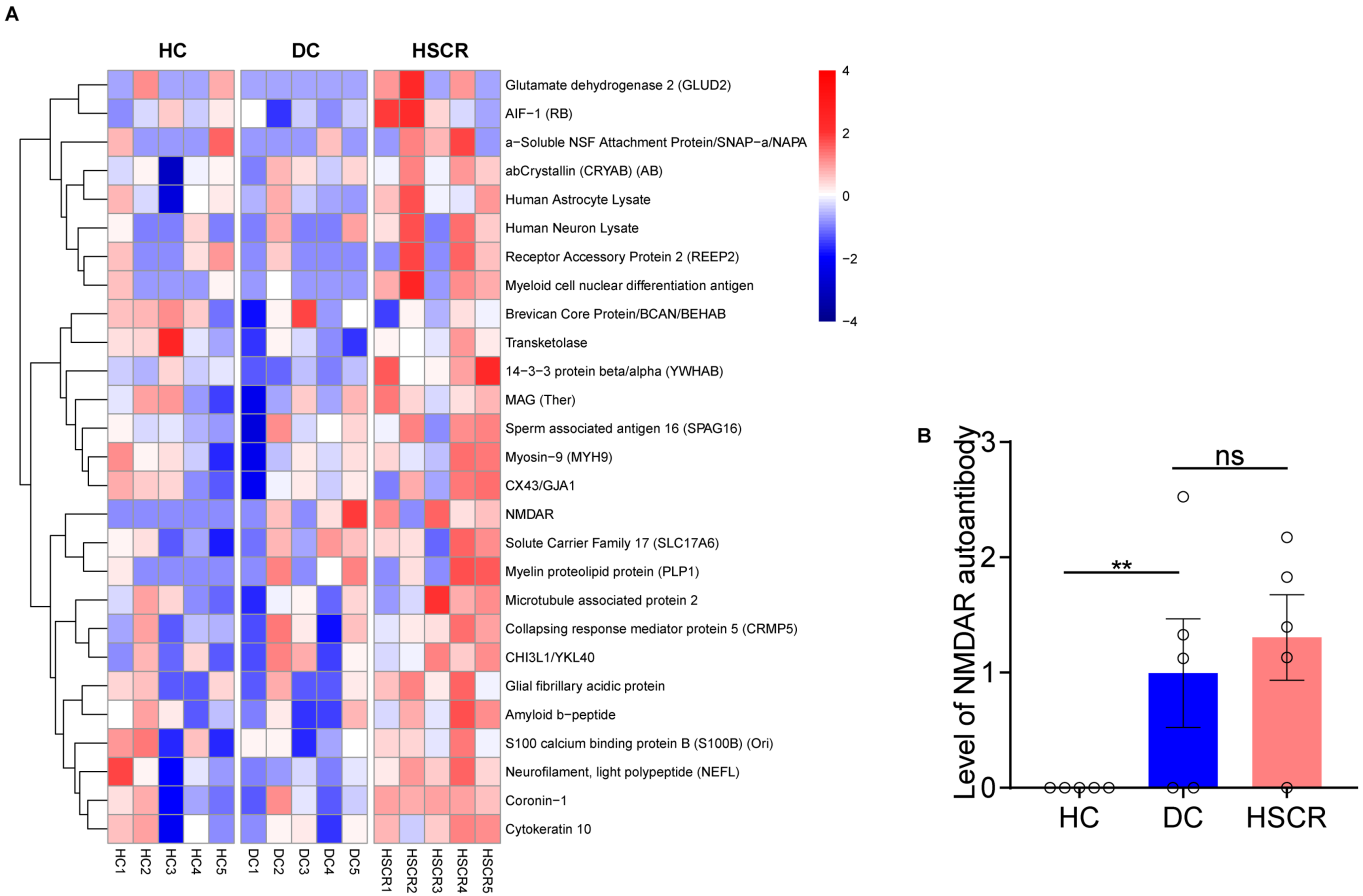
HSCR group had higher plasma NMDAR autoantibody levels than the other control groups. **(A)** The heatmap showed the expression of neural autoantibodies in three groups, respectively [from red (high) to blue (low)]. **(B)** Compare the NMDAR autoantibody level among HSCR, DC, and HC groups based on microarray data. Hirschsprung's disease group (HSCR), other intestinal disease controls (DC) of anal atresia and intestinal stenosis, healthy controls (HC).

Human neural autoantibody microarray cluster analysis (Figure 1A) showed that the expression level of plasma neural autoantibodies significant differences in HSCR, DC and HC group. Compared with the HC group, the HSCR group exhibited significantly higher levels of NMDAR autoantibody (*p* = 0.008). Additionally, HSCR group also showed an upward trend compared with the DC group (Figure 1B).

### Detection of NMDAR autoantibodies in colon tissue by ELISA

**Figure 2:**
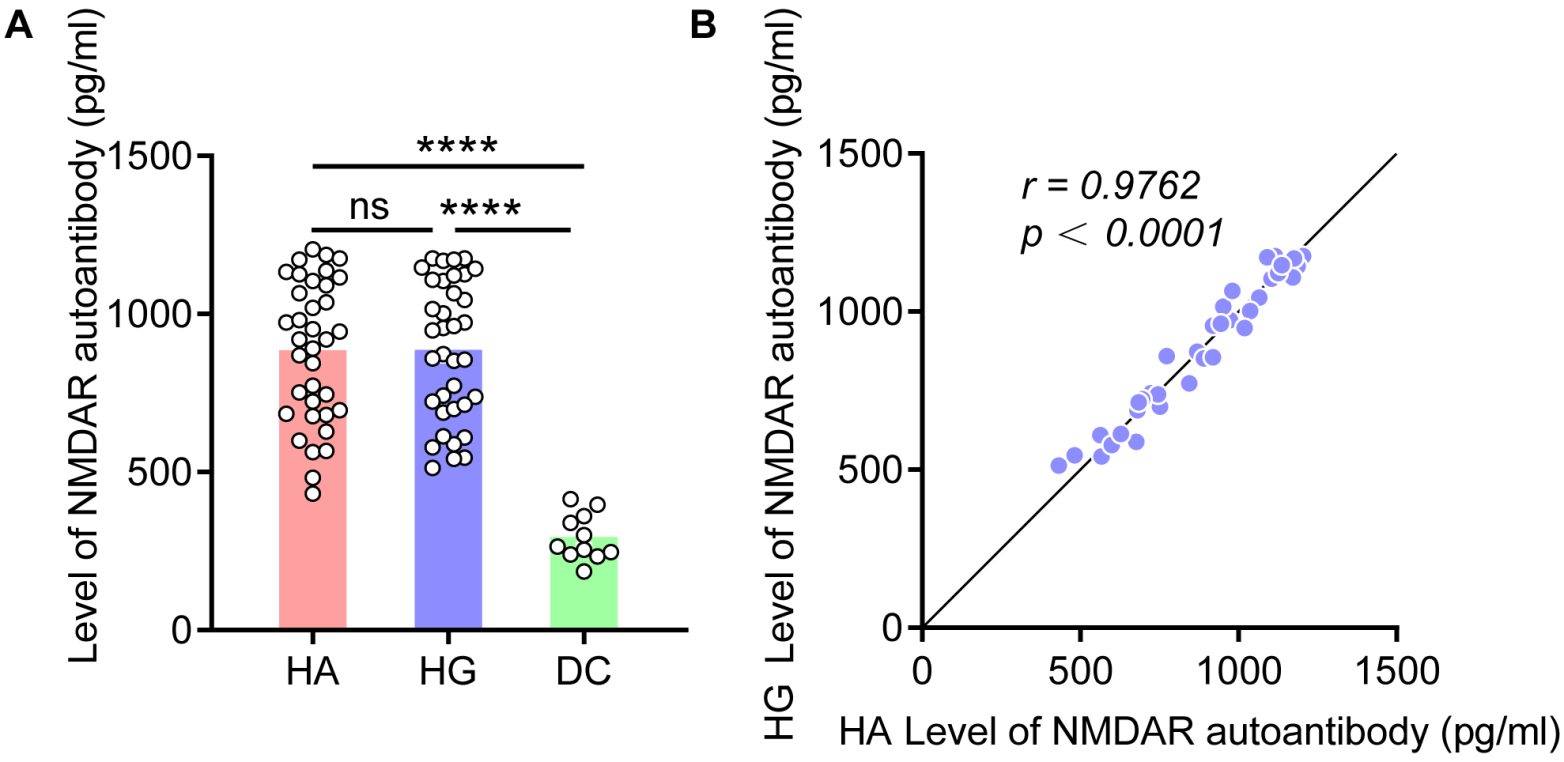
NMDAR autoantibody levels increased in HSCR colon tissue. **(A)** Compare the NMDAR autoantibody level in the colon tissue of HA, HG, and DC. **(B)** Correlation analysis of the expression level of NMDAR autoantibody in the HA and HG of colon tissue. Hirschsprung Disease group (HSCR), HSCR Aganglion (HA), HSCR Ganglion (HG), other intestinal disease controls (DC) of anal atresia and intestinal stenosis. Correlation coefficient (*r*), *p < 0.05, **p < 0.01, ***p < 0.001, ****p < 0.0001.

To explore if there is an increased expression of NMDAR autoantibody in colon tissue, we validated using ELISA testing in independent cohort. The results showed that the expression level of NMDAR autoantibody in HSCR group were higher than those in the DC group, regardless of whether in the HSCR Aganglion (HA, *p* < 0.0001) or HSCR Ganglion (HG, *p* < 0.0001) (Figure 2A). Moreover, when the expression level of NMDAR autoantibody increases in HA, it also shows a similar trend of increase in HG, revealing a highly positive linear correlation (*r* = 0.9762) (Figure 2B).

### Detection of NMDAR autoantibodies in plasma by ELISA

**Figure 3:**
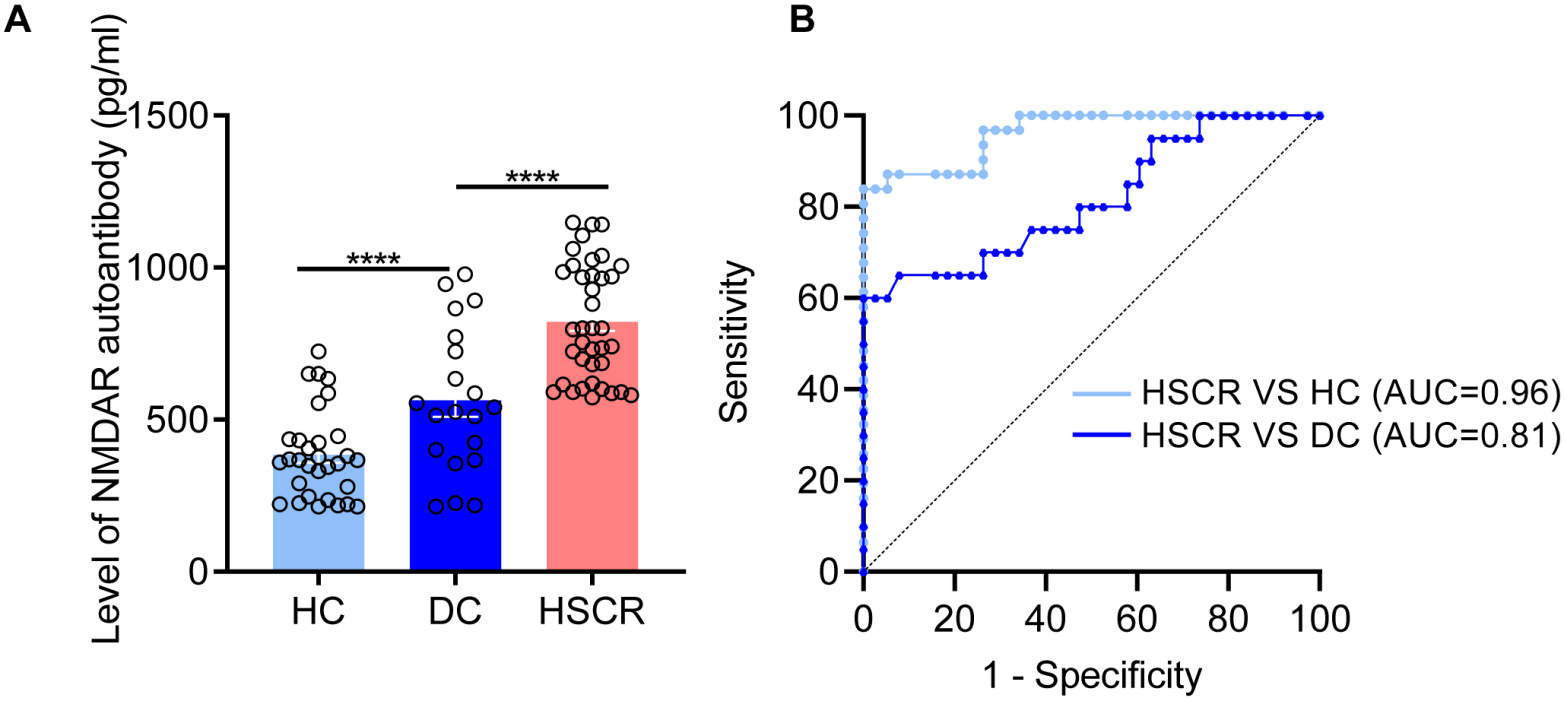
NMDAR autoantibody was identified as a biomarkers for the diagnosis of HSCR. **(A)** Quantitative comparison of plasma NMDAR autoantibody between HSCR group and different control groups (DC, HC). **(B)** The receiver operating characteristic curve is utilized to statistically validate the diagnostic performance of NMDAR autoantibody. Hirschsprung Disease group (HSCR), other intestinal disease controls (DC) of anal atresia and intestinal stenosis, healthy controls (HC). The area under the Receiver Operating Characteristic curve (AUC). *p < 0.05, **p < 0.01, ***p < 0.001, ****p < 0.0001.

In another independent cohort, plasma levels of NMDAR autoantibodies were validated using ELISA, showing significant differences in the HSCR group compared to both the HC (*p* < 0.0001) and DC (*p* < 0.0001) (Figure 3A). Additionally, we assessed the diagnostic performance of the candidate biomarker (Figure 3B). Compared with HC, the plasma NMDAR autoantibody has an AUC of 0.96 in the diagnosis of HSCR (sensitivity 87.1%, specificity 94.74%). While compared with DC, the plasma NMDAR autoantibody has an AUC of 0.81 (sensitivity 70%, specificity 73.68%). These findings further indicate that plasma NMDAR autoantibody can act as a plasma biomarker in the diagnosis of HSCR.

## Discussion

HSCR is a complex intestinal developmental disorder with diverse etiologies. Recent research indicates that autoimmune activation is involved in the progression of HSCR (16). In this study, we utilized a human neuroautoantibody microarray and observed a significant increase in neuronal autoantibodies in the plasma of HSCR patients. Furthermore, we validated that the NMDAR autoantibody in plasma can serve as an objective biomarker for HSCR diagnosis.

BE is one of the most commonly used screening methods for HSCR, with a sensitivity of 70% (64%–76%) and a specificity of 83% (74%–90%) (17). ARM is an effective screening method for HSCR, with a sensitivity of 91% (85%–95%) and a specificity of 94% (89%–97%) in children over six months of age (18). However, it is challenging to achieve the absolute sedation required for neonates (7). Rectal suction biopsy is a diagnostic method for HSCR, requiring the submucosal layer to constitute at least one-third of the sampled tissue. Approximately 9% to 30% of pediatric patients may necessitate repeat biopsies due to insufficient specimens (19). Our study results indicate that plasma NMDAR autoantibody exhibits extremely high accuracy in ruling out healthy individuals, providing a reliable basis for clinical preliminary screening. This can effectively reduce the risk of missed diagnosis, ensuring that patients receive timely attention and further diagnosis. In the disease control group, the expression levels and distribution characteristics of NMDAR autoantibody may change due to the presence of multiple complications or pathological conditions in patients, thereby affecting diagnostic performance. Overall, this diagnostic approach offers a rapid, non-invasive, and efficient alternative for HSCR diagnosis, circumventing the need for pediatric sedation.

NMDAR, a member of the glutamate receptor family, widely expressed in the central nervous system, playing a crucial role in neuronal development and function (20). Studies have shown that NMDAR activation not only stimulates the migration of embryonic cortical neurons (21, 22) but also promotes the proliferation (23–25) and differentiation of neural progenitor cells (26, 27). These processes can be inhibited by NMDAR antagonists. Activation of NMDAR prevents neuronal damage, while NMDAR autoantibodies can downregulate energy metabolism in cultured neurons, leading to neuronal death (28–30). HSCR is caused by migration, proliferation, and differentiation disorders of enteric neural crest cells, with a pathogenesis similar to that of NMDAR autoantibodies-induced neurodevelopmental disorders (31). We found that NMDAR autoantibodies were significantly elevated in the plasma and tissues of the HSCR group, with the highest levels observed in a patient with total colonic HSCR who had a postoperative relapse. Therefore, we speculate whether NMDAR autoantibody is involved in the occurrence of HSCR by inhibiting neuronal migration proliferation, and differentiation, or by inducing neuronal death, and the level of NMDAR autoantibody may be related to the severity and recurrence of HSCR. Interestingly, we observed nearly identical NMDAR autoantibody levels in the HSCR HA and HG. This finding may suggest that the NMDAR autoantibody response is not solely dependent on the presence or absence of ganglia in the affected tissue. One possible explanation could be the presence of upstream regulatory mechanisms that influence NMDAR autoantibody production in both aganglionic and ganglionic segments of the bowel. Additionally, this similarity in autoantibody levels might reflect the systemic nature of the immune response in HSCR patients, which is not limited to the aganglionic segment alone. Further studies are warranted to explore these hypotheses and to elucidate the precise role of NMDAR autoantibody in the pathogenesis of HSCR.

### Study limitations

This study has several limitations. Firstly, differences in neonatal history, sample size, methods of sample collection, and environmental factors between cases and controls may potentially affect the levels of NMDAR autoantibody, which is indeed a significant factor to be considered in study design. To minimize the potential confounding factors, it is necessary to strictly control these variables in the experimental design and conduct prospective studies for further validation.

Secondly, this study was conducted at a single center. Although NMDAR autoantibody demonstrated high diagnostic accuracy in distinguishing the HSCR group from the HC group, their diagnostic efficacy was moderate when comparing the HSCR group with the DC group. To assess the diagnostic accuracy of NMDAR autoantibody and to exclude potential selection bias, future studies should involve multicenter, large-sample clinical cohorts.

Ultimately, we have not further elucidated whether NMDAR autoantibody participates in the occurrence and development of HSCR, or if targeting these autoantibodies could serve as a treatment for HSCR, which will be the focus of future investigations.

## Conclusions

In summary, our research has identified and provided preliminary evidence for that NMDAR autoantibody is a highly effective plasma biomarker for diagnosing HSCR. Furthermore, it may be implicated in the pathogenesis of HSCR.

## Data Availability

All relevant data is contained within the article: The original contributions presented in the study are included in the article/Supplementary Material, further inquiries can be directed to the corresponding author.
