# Supplementary material for: Plasma NMDAR autoantibody: a new biomarker for the diagnosis of Hirschsprung disease
Source: Front Pediatr. 2025 Feb 21;13:1514323. doi: 10.3389/fped.2025.1514323 (PMC11885489; doi:10.3389/fped.2025.1514323)
Supplement: Supplementary file 1 [file Table1.docx]

**[Supplementary material](file:///F:\\研究生资料\\2024.03.21\\第六版投稿\\Archives%20of%20Disease%20in%20Childhood-Fetal%20and%20Neonatal%20Edition\\5.28%20Plasma%20NMDAR%20Autoantibody%20a%20New%20Biomarker%20for%20the%20Diagnosis%20of%20Hirschsprung%20disease.docx#/#)**

**Table 1. Basic characteristics of microarray analysis research objects.**

| Characteristic | Disease | control | | *p* Value |
| --- | --- | --- | --- | --- |
|  | HSCR  (n=5) | DC  (n=5) | HC  (n=5) |  |
| Age,month | 8.8±6.42 | 6.8±1.92 | 10±8.3 | ＞0.05 |
| Male | 4 (80%) | 4 (80%) | 4 (80%) | ＞0.05 |

**Table 2. Basic characteristics of colon tissue sample research subjects**.

| Characteristic | HSCR  (n=36) | DC  (n=11) | *p* Value |
| --- | --- | --- | --- |
|  |  |  |  |
| Age,month | 5 (4-11) | 6 (5-7) | ＞0.05 |
| Male | 29(80.5%) | 8 (72.7%) | ＞0.05 |

**Table 3. Basic characteristics of plasma sample study subjects**.

| Characteristic | Disease | control | | *p* Value |
| --- | --- | --- | --- | --- |
|  | HSCR  (n=38) | DC  (n=20) | HC  (n=31) |  |
| Age, month | 7(5-12) | 6 (5-10.5) | 6 (4-6.5) | ＞0.05 |
| Male | 27(71%) | 14 (70%) | 22 (71%) | ＞0.05 |

Table 1 to 3 Notes: Age in HSCR group represented the age of onset, and the data was represented as mean ± standard deviation if it follows a normal distribution. If not following a normal distribution, the data was represented as median and interquartile range. Gender data presented as the number of male cases and the percentage of males. [Hirschsprung Disease](file:///F:\\研究生资料\\2024.03.21\\第六版投稿\\2024.05.21%20EXPERT%20REVIEW%20OF%20MOLECULAR%20DIAGNOSTICS投稿版.docx#/#) group (HSCR), other intestinal disease controls (DC) of anal atresia and intestinal stenosis, healthy controls (HC). Statistical significance is noted when *p* < 0.05.
